# Metformin Affects Olaparib Sensitivity through Induction of Apoptosis in Epithelial Ovarian Cancer Cell Lines

**DOI:** 10.3390/ijms221910557

**Published:** 2021-09-29

**Authors:** Patrycja Gralewska, Arkadiusz Gajek, Agnieszka Marczak, Aneta Rogalska

**Affiliations:** Department of Medical Biophysics, Faculty of Biology and Environmental Protection, Institute of Biophysics, University of Lodz, 90-236 Lodz, Poland; patrycja.gralewska@edu.uni.lodz.pl (P.G.); arkadiusz.gajek@biol.uni.lodz.pl (A.G.); agnieszka.marczak@biol.uni.lodz.pl (A.M.)

**Keywords:** combination therapy, metformin, olaparib, ovarian cancer, PARP inhibitor, replication stress

## Abstract

This study examined the effect of combination treatment with the poly (ADP-ribose) polymerase inhibitor olaparib and metformin on homologous recombination (HR)-proficient epithelial ovarian cancer (EOC). Ovarian cancer cell lines (OV-90 and SKOV-3) were treated with olaparib, metformin, or a combination of both. Cell viability was assessed by MTT and colony formation assays. The production of reactive oxygen species (ROS) and changes in mitochondrial membrane potential were examined using the specific fluorescence probes, DCFH2-DA (2′,7′-dichloro-dihydrofluorescein diacetate) and JC-1 (5,5′,6,6′-tetrachloro-1,1′,3,3′-tetraethylbenzimidazolcarbocyanine). Apoptotic and necrotic changes were measured by double staining with Hoechst 33258 and propidium iodide, orange acridine and ethidium bromide staining, phosphatidylserine externalization, TUNEL assay, caspase 3/7 activity, and cytochrome c and p53 expression. Compared with single-drug treatment, the combination of olaparib and metformin significantly inhibited cell proliferation and colony formation in HR-proficient ovarian cancer cells. ROS production preceded a decrease in mitochondrial membrane potential. The changes in ROS levels suggested their involvement in inducing apoptosis in response to combination treatment. The present results indicate a shift towards synergism in cells with mutant or null p53, treated with olaparib combined with metformin, providing a new approach to the treatment of gynecologic cancers. Taken together, the results support the use of metformin to sensitize EOC to olaparib therapy.

## 1. Introduction

Ovarian cancer is one of the most lethal gynecologic malignancies worldwide [1,2] with a five-year survival rate of <40% [3]. The majority of women with recurrent ovarian cancer have BRCA wild-type tumors (BRCA^WT^), which are difficult to treat and are associated with poor outcomes [4]. Most type II ovarian carcinomas (96%) have the TP53 mutation [5,6]. Cytoreductive surgery and platinum–taxane combination chemotherapy remains the primary treatment for epithelial ovarian cancer (EOC); however, most patients with EOC develop platinum resistance [7].

Metformin, a biguanide derivative that is commonly used as a hypoglycemic agent, inhibits cell proliferation in several human malignancies, including pancreatic, thyroid, gastric, endometrial, and ovarian cancers [8,9]. It demonstrated significant synergy with chemotherapeutic drugs in both in vitro and in vivo models, including cisplatin, gefitinib [10], sorafenib [11], everolimus, or trastuzumab. Metformin induces apoptosis and inhibits angiogenesis and the metastatic spread of ovarian cancer [12]. This drug activates mitochondrial oxidase and increases the level of reactive oxygen species (ROS) within the mitochondrial matrix by inhibiting complex 1 of the mitochondrial electron transport chain [13]. Metformin treatment in women with type 2 diabetes prevents ovarian cancer occurrence. Metformin-treated patients show increased five-year survival rates. In addition, metformin eliminates cancer stem cells, thus contributing to a better prognosis in ovarian cancer patients [14].

Poly (ADP-ribose) polymerase (PARP) is a superfamily of enzymes with different cellular functions, including cell-cycle regulation, inflammation, and DNA repair. PARP inhibitors (PARPis) increase replication stress and genome instability, thereby leading to cell death [15]. PARPs are key components of base excision repair, which involves the recruitment of repair enzymes to the site of single-stranded DNA breaks [16]. The first PARPi approved by the US Food and Drug Administration (FDA) was olaparib (Lynparza). In 2018, the FDA approved olaparib for the maintenance treatment of patients with BRCA mutated (BRCA^MUT^) advanced EOC who demonstrated complete or partial response to first-line platinum-based chemotherapy [17]. Clinical trials show that when PARPis are used to treat ovarian cancer with BRCA mutations, the risk of death or disease progression decreases by 70–75%. Clinical trials #19 and SOLO evaluated the efficacy of olaparib, ARIEL3 evaluated rucaparib, VELIA evaluated veliparib, and PRIMA and ENGOT-OV16/NOVA focused on niraparib. Although some trials expanded the use of PARPis beyond BRCA mutation status, the effectiveness of PARPis may be limited to certain patient populations with BRCA 1/2 mutation [18,19,20]. PARPis may also benefit patients who are sensitive to platinum-based chemotherapy or have homologous recombination (HR) deficiency caused by mutations other than those in the BRCA 1/2 genes [21]. Acquired resistance to platinum and PARPis in EOC is associated with reversion of mutated BRCA genes and the restoration of homologous recombination repair functions [22,23,24].

Studies suggest that PARP is an effective target for synthetic lethality, a phenomenon in which the inactivation of either of two genes is nonlethal, whereas the simultaneous inactivation of both genes is lethal [25,26]. However, a high percentage of patients with EOC do not respond to PARPis. The successful treatment of cancer depends on the type of cell death, and the favorable form of death is apoptosis. Apoptosis is induced by an imbalance between pro- and antiapoptotic protein regulators. We therefore focused our investigation on the mechanism of induction of apoptosis after combined treatment with metformin and PARPi in HR-proficient ovarian cancer cells [27,28,29,30,31].

In the current study, we tested whether the addition of metformin to olaparib could potentiate the effects of the PARPi and increase cell death in BRCA^WT^ EOC with mutated and null p53. Searching for effective treatment strategies is critical to improve the therapeutic outcomes and median overall survival of this patient population.

## 2. Results

### 2.1. Olaparib and Metformin Reduce the Viability of Ovarian Cancer Cells

**Figure 1:**
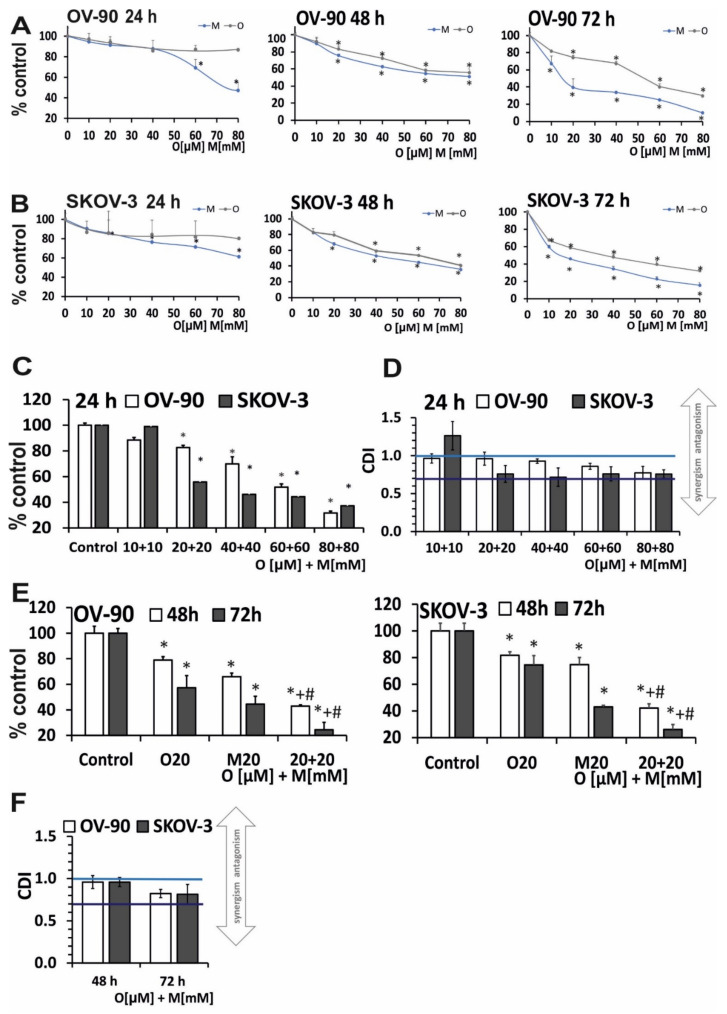
Metformin in combination with olaparib decreased cell viability more effectively than each single agent. (**A**,**B**) Cell viability after treatment for 24–72 h with olaparib (10–80 µM) and metformin (10–80 mM) in OV-90 and SKOV-3 cells assessed by the MTT assay. (**C**) The combination effect of olaparib (10–80 µM) and metformin (10–80 mM) was evaluated by the MTT assay after treatment for 24 h. (**D**) Drug interaction analysis based on the MTT assay after treatment for 24 h. In both cell lines, the combination of O (20 µM) + M (20 mM) showed a synergistic effect (SKOV-3, CDI = 0.74; OV-90 CDI = 0.94). (**E**) The effect of combination treatment with olaparib (20 µM) and metformin (20 mM) evaluated by the MTT assay after treatment for 48–72 h. (**F**) Drug interaction analysis based on the MTT assay CDI value for combination of O (20 µM) + M (20 mM) after treatment for 48 h (SKOV-3, CDI = 0.82; OV-90, CDI = 0.95) and for 72 h (SKOV-3, CDI = 0.69; OV-90, CDI = 0.81). Data represent the mean ± SD of three biologic assays. * *p* < 0.05 for olaparib, metformin, or a combination of both drugs vs. control cells; # *p* < 0.05 for olaparib vs. combination (O + M); + *p* < 0.05 for metformin vs. combination (O + M).

The antiproliferative activity of the compounds was assessed using the MTT assay. To discriminate between early and late effects of olaparib and metformin, cell lines were exposed to increasing drug concentrations for 24–72 h. Olaparib did not markedly decrease cell survival rate after 24 h. A similar response was observed in both cell lines. Metformin was more cytotoxic towards ovarian cancer cells than olaparib and caused a dose-dependent decrease in cell viability; however, attention should be paid to the differences of an order of magnitude between the doses of the drugs used. Olaparib reduced the viability of OV-90 cells to 86.8% and metformin to 47.2%. In SKOV-3 cells, olaparib decreased viability to 82.64%, and metformin to 49.56%, as shown in Figure 1A,B. An additional decrease in the viability of OV-90 cells by about 30% after olaparib treatment was achieved by extending the incubation time to 48 h (Figure 1E,F). SKOV-3 cells showed a 40% decrease in viability after olaparib and a 14% decrease after metformin treatment for 24 h. The results indicate marked differences in the effects of olaparib and metformin between 72 and 48 h. As shown in Figure 1E,F, olaparib reduced the viability of OV-90 cells to 30% and metformin reduced viability to 9.8%. In SKOV-3 cells exposed to the indicated concentrations of the investigated drugs, the viability was reduced to a lesser degree than in OV-90 cells. The viability in SKOV-3 cells decreased after 72 h of incubation to 31.7% and 15.4% for olaparib and metformin, respectively. A comparison of cell viability after 24–72 h of treatment with olaparib and metformin revealed that the duration of treatment is more important for the cytotoxic effects of olaparib than for those of metformin.

Combined treatment with olaparib and metformin for 24 h significantly decreased viability at 20 µM olaparib and 20 mM metformin and at higher concentrations in both cell lines (Figure 1C). Thus, for further experiments, the lowest effective concentration pair was selected. We distinguished between low (blue line, CDI < 1) and strong (dark blue line, CDI < 0.7) synergism in Figure 1. In both cell lines, the combination of O (20 µM) + M (20 mM) was synergistic (SKOV-3, coefficient of drug interaction (CDI) = 0.74; OV-90, CDI = 0.94) (Figure 1D). The studies also confirmed beneficial effect at the selected concentration after longer incubation times (48 h: SKOV-3, CDI = 0.82; OV-90 CDI = 0.95; 72 h: SKOV-3, CDI = 0.69; OV-90 CDI = 0.81, Figure 1F).

**Figure 2:**
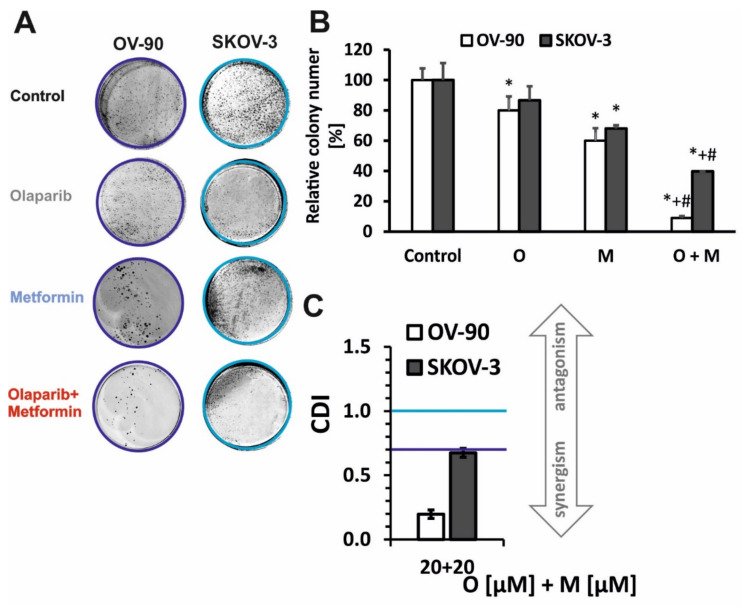
Prolonged incubation with metformin in combination with olaparib decreased colony numbers (**A**,**B**). Colony formation was evaluated following treatment with 20 µM olaparib, 20 mM metformin, or combination treatment with olaparib (20 µM) and metformin (20 mM) in SKOV-3 and OV-90 cells. (**C**) Drug interaction analysis based on colony formation assay. Data represent the mean ± SD of three biologic assays. * *p* < 0.05 for olaparib, metformin, or a combination of both drugs vs. control cells; # *p* < 0.05 for olaparib vs. combination (O + M); + *p* < 0.05 for metformin vs. combination (O + M).

The long-term effects of single-drug (olaparib, metformin) or combined-drug treatment on ovarian cancer cell lines were evaluated in vitro by performing a colony-formation assay (Figure 2A,B). The colony-forming ability after treatment with 20 µM PARPi and 20 mM metformin was comparable between the two cell lines. Combination treatment with O+M decreased colony formation to 39.7% in SKOV-3 (CDI = 0.67) and to 9.01% in OV-90 cells (CDI = 0.196) (Figure 2C). In contrast to the results of the viability assessment after 24 h of incubation, the OV-90 line was more sensitive to the action of the combined treatment.

### 2.2. Treatment with Olaparib and Metformin Leads to Increased Oxidative Stress

The effect of metformin or olaparib alone and in combination on the production of ROS was examined. The results showed that ROS levels depended on the cell line and the treatment duration.

**Figure 3:**
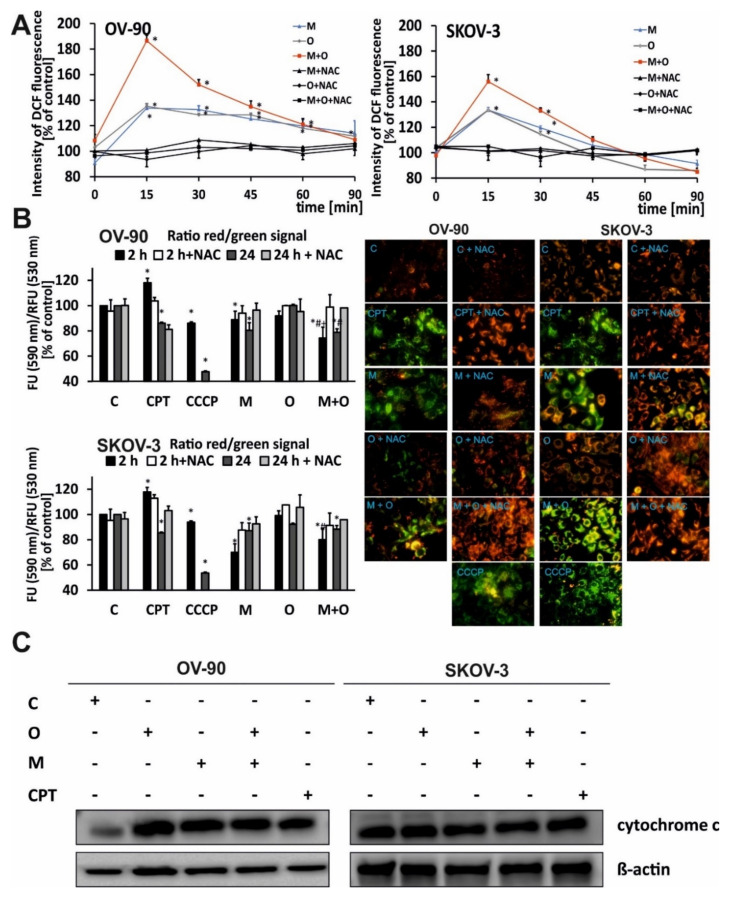
Metformin combined with olaparib induced oxidative stress and decreased the mitochondrial membrane potential. (**A**) The kinetics of ROS generation in OV-90 and SKOV-3 cells treated with O (20 µM), M (20 mM), or O (20 µM) + M (20 mM) were measured for up to 90 min in the presence or absence of an antioxidant (NAC); C: control, CPT: Camptothecin. (**B**) The fluorescence ratio of JC-1 dimers/JC-1 monomers in the control was assumed to be 100%. OV-90 and SKOV-3 cells were stained with the fluorescence probe JC-1 after 24 h of incubation with O (20 µM), M (20 mM), or O (20 µM) + M (20 mM) or carbonyl cyanide m-chlorophenyl hydrazone (CCCP) (10 µM), or CPT (5 µM). (**C**) Cytochrome c and β-actin expression was analyzed by western blotting. All data are from three biologic assays and are graphed as the mean ± SD. * *p* < 0.05 for olaparib, metformin, or a combination of both drugs vs. control cells; # *p* < 0.05 for olaparib vs. com-bination (O + M); + *p* < 0.05 for metformin vs. combination (O + M).

The cellular DCF (2′,7′-dichlorofluorescein) fluorescence intensity was lower in cells incubated with each drug alone than in cells treated with the drug combination. The combination of olaparib and metformin had a stronger effect on ROS generation than each drug alone. The greatest enhancement of ROS production was observed after 15 min of combination treatment. The highest fluorescence intensity of the probes was 156% and 186% of starting value in SKOV-3 and OV-90 cells, respectively. After an initial sharp increase in ROS level, a gradual decrease to almost control level occurred during the next 90 min, as shown in Figure 3A. This suggests that intracellular antioxidant systems neutralized ROS.

When both cell lines were treated with single drugs, the highest fluorescence intensity, indicating the presence of ROS, was also observed after 15 min of incubation. Both metformin and olaparib alone induced similar (~35%) levels of ROS in SKOV-3 or OV-90 cancer cells. However, the kinetics of this process was different and cell-dependent. In OV-90 cells, the decrease in the level of ROS after incubation was slower and achieved 120% of starting value after 60 min. In SKOV-3 cells, after 90 min of incubation, ROS levels decreased to ~98% for metformin and to ~86% for olaparib. In both cell lines, pre-incubation with NAC protected cells from ROS, decreasing its amount to the control level (Figure 3A).

### 2.3. The Combination of Metformin and Olaparib Modulate the Release of Cytochrome c and Causes loss of Mitochondrial Membrane Potential

The impact of metformin, olaparib, and combination treatment on the mitochondrial membrane potential (ΔΨm) in OV-90 and SKOV-3 cells was assessed using fluorimetric analysis after staining with the fluorescent dye 5,5′,6,6′-tetrachloro-1,1′,3,3′- tetraethylbenzimidazolcarbocyanine (JC-1) (Figure 3B). The red fluorescence of JC-1 dimers (high mitochondrial potential) was observed in control cells. Drug treatment dramatically increased the green fluorescence of JC-1 monomers, indicating a decrease in mitochondrial membrane potential. The highest green fluorescence intensity was observed in OV-90 cells treated with the drug combination. In SKOV-3 cells, strong green fluorescence of JC-1 monomers was observed after treatment with metformin and drug combination. In probes preincubated with the antioxidant NAC, the fluorescence was similar between treated cells and control cells. All drugs induced time-dependent changes. The rate of JC-1 probe fluorescence in OV-90 cells decreased by 11% after 2 h of incubation with metformin, whereas no changes were observed in response to olaparib. Drugs used in combination caused the highest decrease in the ΔΨm (26% after 2 h of treatment and 22% after 24 h). SKOV-3 cells were more sensitive to metformin regarding ΔΨm changes. However, the effect did not differ between single and combination treatment regardless of the incubation time. chlorophenylhydrazone (CCCP), an uncoupler of oxidative phosphorylation, was used as a positive control for depolarization of the ΔΨm and cells were treated for 30 min before staining.

The disruption of mitochondrial integrity is a key event in apoptosis. OV-90 and SKOV-3 cells were treated with O (20 µM), M (20 mM,) O (20 µM) + M (20 mM), or CPT (5 µM) for 24 h. The combination of metformin and olaparib upregulated cytochrome c to a greater extent than single treatment with metformin or olaparib (Figure 3C), and this effect was stronger in SKOV-3 cells. PARP inhibition led to a 1.2-fold increase in the level of cytochrome c in SKOV-3 and a 3.8-fold increase in OV-90 cells. Metformin upregulated cytochrome c by 1.2-fold in SKOV-3 and 4.3-fold in OV-90 cells, whereas combination treatment upregulated cytochrome c by 3.8-fold, the same as olaparib, in OV-90 cells. In SKOV-3 cells, combination of drugs caused a 2-fold increase compared with the control. Histograms with a fold changes of cytochrome c expression are shown in Figure S1.

### 2.4. Metformin Increase the Sensitivity of Ovarian Cancer Cells to Olaparib—Assessment of Apoptosis and Necrosis by Double Staining

**Figure 4:**
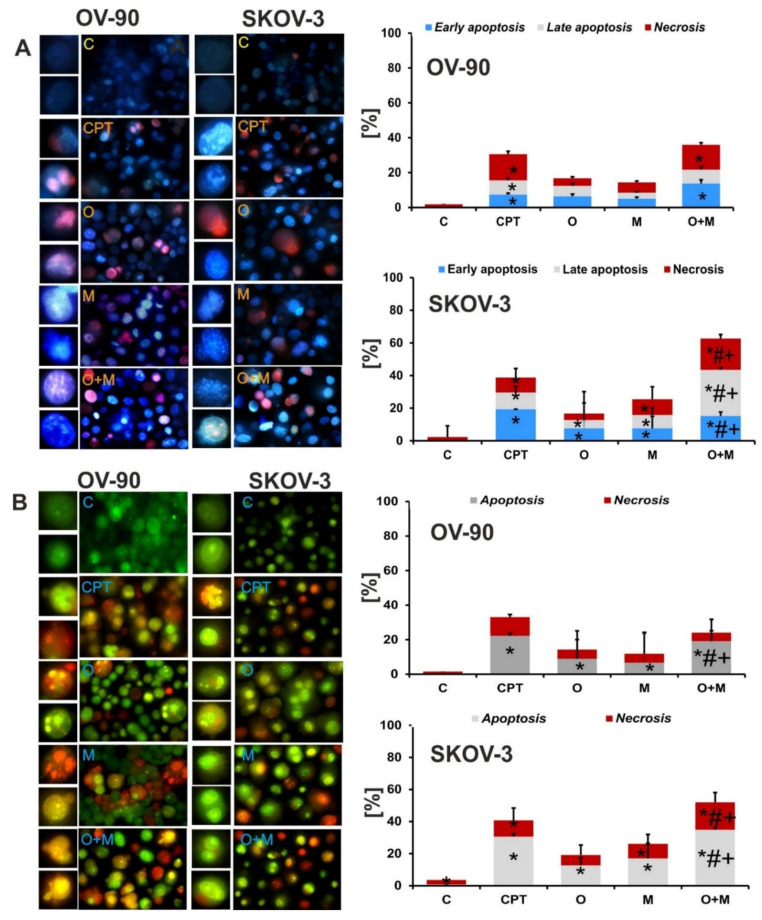
Combination treatment caused a greater cell death in both tested cell lines compared with each drug alone. Representative images of the apoptotic and necrotic changes caused by the compounds; C: control. The cells were visualized under a fluorescence microscope (Olympus IX70). (**A**) Apoptotic and necrotic changes visualized after double staining with Hoechst 33258/propidium iodide. (**B**) Apoptotic and necrotic changes visualized with acridine orange and ethidium bromide staining. Data are from three biologic assays and are graphed as the mean ± SD. * *p* < 0.05 for olaparib, metformin, or a combination of both drugs vs. control cells; # *p* < 0.05 for olaparib vs. combination (O + M); and + *p* < 0.05 for metformin vs. combination (O + M).

To determine the type of cell death induced by metformin and olaparib in ovarian cancer cells, the morphological changes of the cells were analyzed after double staining with Hoechst 33258/propidium iodide and acridine orange/ethidium bromide. Generally, apoptotic changes were greater in SKOV-3 cells. Combination treatment caused an almost 2-fold higher increase in the fraction of early apoptotic cells than each drug alone. Similarly, the percentage of late apoptotic cells was also significantly higher after combination treatment (28.4% for combination treatment vs. 5.1% for olaparib and 8.2% for metformin). In the OV-90 cell line, the changes were not as pronounced as in the SKOV-3 cell line, and the percentages of early and late apoptotic and necrotic cells of each investigated fraction after the combined treatment were lower at 13.8%, 7.9%, and 14.2%, respectively. The results are presented in Figure 4A.

Double staining with acridine orange and ethidium bromide allowed the detection of the morphological features of apoptosis such as chromatin condensation and nuclear fragmentation, as well as alterations in the size and the shape of the cells. Changes were observed mainly after the combined treatment with olaparib and metformin (Figure 4B). Swollen, red fluorescent cells, which are characteristic of necrotic cell death, were also visible. The significant increase in the number of apoptotic and necrotic cells after combination treatment, compared with each drug alone implies that combination treatment had a greater cytotoxic effect in both tested cell lines. The results are in accordance with those obtained by double staining with Hoechst 33258 and propidium iodide.

### 2.5. Metformin in Combination with Olaparib Increases Caspase 3/7 Dependent DNA Damage and Phosphatidylserine Externalization in Ovarian Cancer Cells

To evaluate the possible role of apoptosis features after olaparib or metformin treatment as well as their combination, the mode of cell death triggered by these drugs was investigated using Annexin V, TUNEL, and caspase3/7 assays. Measurement of phosphatidylserine (PS) externalization, revealed that OV-90 cells were more susceptible to changes in the cell membrane than SKOV-3 cells. Generally, metformin monotherapy increased Annexin fluorescence intensity to a greater extent than olaparib. In the SKOV-3 cell line, the intensity of Annexin fluorescence was >2-fold higher in the samples incubated with the two drugs (7.67) than in those treated with olaparib (2.52), which showed significantly lower fluorescence than those treated with metformin (6.07). Similar differences in Annexin fluorescence intensity between olaparib (11.45) and metformin monotherapy (20.16) were observed in the OV-90 cell line. However, cotreatment strategies resulted in a significant increase in PS externalization (21.01) only when compared with olaparib treatment, and these changes were not statistically significant compared with metformin monotherapy (20.15).

**Figure 5:**
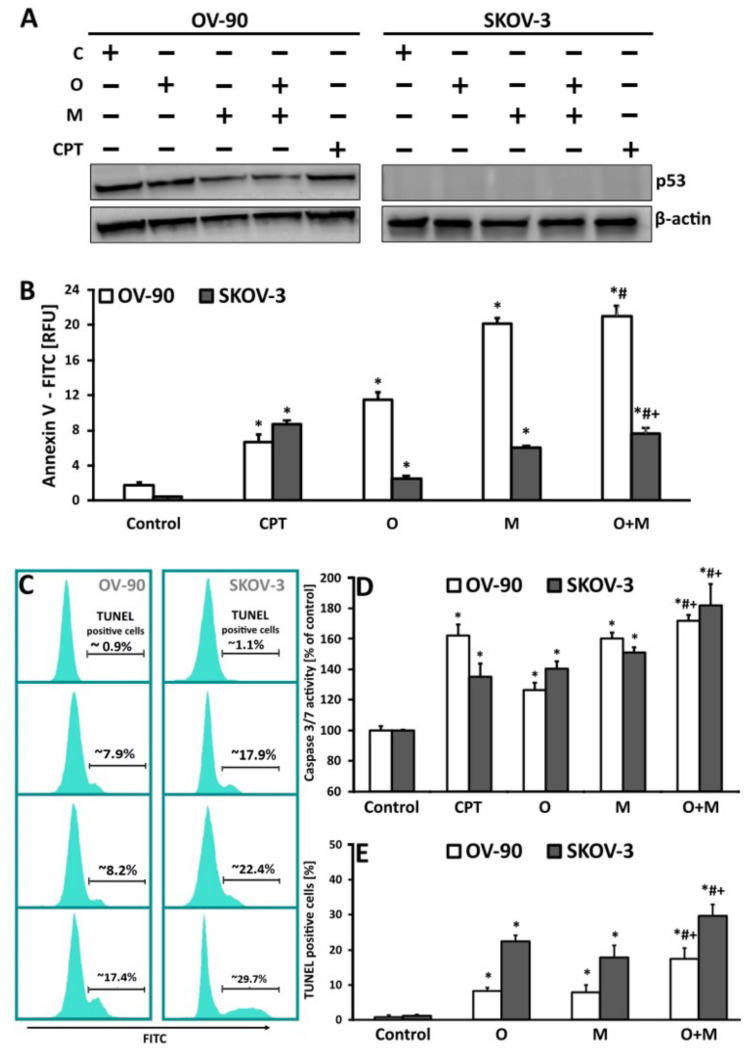
DNA damage and the rate of apoptosis induced by olaparib, metformin, and their combination in OV-90 and SKOV-3 cells. (**A**) The cells were treated with O (20 µM), M (20 mM), or O (20 µM) + M (20 mM) with or without CPT (5 µM) and lysates were collected at 24 h. p53 and β-actin expression was analyzed by western blotting. SKOV-3 has no p53 activity. (**B**) After 24 h of drug treatment, the intensity of Annexin fluorescence was measured in cells with exposed PS. The results are presented as relative fluorescence units [RFU]. (**C**) Representative histograms obtained for samples treated with metformin or olaparib and its combination. The position of the marker representing TUNEL-positive cells (with fragmented DNA) was adjusted according to the negative control. (**D**) Changes in the activity of caspase 3/7 after exposure of SKOV-3 and OV-90 cell lines to metformin or olaparib and their combination for 24 h. The results are presented as a percentage activity of caspase 3/7, where the fluorescence value of the un-treated control was taken as 100%. (**E**) The effect of metformin, olaparib, and their combination on the induction of DNA damage in the SKOV-3 and OV-90 cancer cell lines, as estimated by the TUNEL assay. All data are from the three biologic assays and are graphed as the mean ± SD. * *p* < 0.05 for olaparib, metformin, or a combination of both drugs vs. control cells; # *p* < 0.05 for olaparib vs. combination (O + M); + *p* < 0.05 for metformin vs. combination (O + M).

An important feature of apoptotic cells used for the detection of programmed cell death is DNA fragmentation into sections equal to the multiples of the length of nucleosomes (180–200 bp) [32]. TUNEL-positive cells with the 3′-hydroxyl termini of DNA strand breaks were observed in the SKOV-3 and OV-90 cell lines after drug treatment. Drug combination caused the most pronounced increase in apoptotic cells (approximately 30%) in the SKOV-3 cell line after the 24 h of incubation. At the same time point, a considerably lower number of TUNEL-positive events was detected in cells treated with olaparib or metformin alone at approximately 22% and 18%, respectively (Figure 5). The OV-90 cell line was significantly more resistant to apoptosis induction by the drugs than the SKOV-3 cell line. The percentage of cells with 3′-hydroxyl termini of damaged DNA after metformin treatment (7.9%) did not differ markedly from that in olaparib-treated cells (8.2%). The drug combination also induced significant changes in the OV-90 cells (about 17.4%). Comparison of the viability and the apoptotic, necrotic and total cells death in SKOV-3 and OV-90 cell lines after 24 h treatment with metformin (20 mM), olaparib (20 µM) and their combination (20 mM + 20 µM) is shown in Table S1.

Caspases are key mediators of DNA fragmentation. Caspase 3/7 activation, independently of the type of drug, achieved the maximal level after 24 h of treatment in both cell lines. Generally, the increase in caspase 3/7 activity was higher in samples exposed to the drug combination than in those treated with metformin or olaparib alone. The greatest increase in caspase activation was observed with metformin treatment and did not exceed 60% in OV-90 cells and 50% in SKOV-3 cells. A slight increase in caspase activity (by 26% in OV-90 and 40% in SKOV-3) was observed in cells treated with olaparib alone. Activation of cysteine proteases after treatment with the drug combination was considerably greater in SKOV-3 cells than in OV-90 cells (82% vs. 70%) after 24 h of treatment. Taken together, these findings suggest that SKOV-3 cells were more susceptible to apoptosis induced by the drug combination than OV-90 cells (Figure 5D). Changes in caspase 3/7 activity in SKOV-3 and OV-90 cells treated with metformin or olaparib and their combination for 24 h in the presence of the caspase inhibitor, Z-FA-FMK are shown in Figure S2.

Cells were treated with a caspase-3/-7 activity inhibitor to confirm that the enzymes activated by the compounds were cysteine proteases. The inhibitors caused a decrease in the activity to a level observed in the control samples, indicating that the reactions were specific and resulted from the activity of the tested compounds (data not shown).

The effect of the treatment on a key tumor suppressor protein, p53, was measured by western blotting, as shown in Figure 5A. The cells were treated with O (20 µM), M (20 mM), O (20 µM) + M (20 mM) or CPT (5 µM) and lysates were collected at 24 h. In the OV-90 cell line, which carries a p53 mutation, combination treatment led to a 3.3-fold decrease in the level of p53, whereas olaparib and metformin alone caused a 1.3-fold and 2-fold decrease, respectively, compared with the control. SKOV-3 has no p53 activity, as it lacks a TP53 functional gene. Histograms with a fold changes of p53 expression are shown in Figure S1.

## 3. Discussion

Targeted therapies have achieved good results in many types of cancer, although their efficacy is limited by problems such as toxicity and resistance. To address these limitations, the combined use of known drugs has drawn considerable attention. Olaparib (PARPi) was approved as a first-line maintenance therapy for BRCA^MUT^ advanced ovarian cancer; however, only a small percentage of EOC (~15%) cases are BRCA-mutated, and resistance to olaparib is common. Most patients with high-grade serous EOC exhibit the phenotype of defective HR without BRCA mutations known as “BRCAness” [33]. Metformin is a low-toxicity therapeutic approach that targets various key pathways and is crucial for cancer treatment [34]. Metformin has been used for over 60 years, whereas olaparib is a relatively new drug that has been used as part of chemotherapy since the beginning of 2015. Metformin and olaparib have high antineoplastic activity against advanced and metastatic ovarian cancer types resistant to anthracyclines, which has opened new possibilities for the treatment of these tumors. In clinical trials, PARPis significantly increased the efficacy of standard therapies through combination treatment with carboplatin (NCT01033292), cediranib (NCT02681237), or nivolumab (NCT03522246). In breast cancer cells, metformin induces cell death via PARP activation and apoptosis [33], and in ovarian cancer, it induces cell death in combination with other drugs such as cisplatin [34,35,36].

It should be noted that cell-culture conditions may be an important determinant of metformin resistance in the treatment of some cancers. Cancer cells having different metabolic phenotypes, show clear differential response to metformin treatment based on glucose concentration [37]. Recent data showed that low glucose (2.5mM) in culture medium enhances sensitivity of SKOV-3 cells to metformin. These results suggested that low glucose and metformin induce cell apoptosis through triggering ER stress, which is associated with the ROS/ASK1/JNK pathway. Moreover, ASK1 activation was involved in the loss of mitochondrial membrane potential, caspase 3 cleavage and the subsequent release of cytochrome c [38]. As a rule, cell culture media contain up to 55 mM glucose, while typical plasma levels are ranged from 5 to 7 mM. Higher than 10 mM represents a “diabetic” concentration. In our work, both cell lines were cultured in media with glucose levels above 10 mM, representing more metformin resistance conditions. The aim of this study was to determine the effectiveness of combination treatment with olaparib and metformin in a research model representing an oncological ovarian disease. There is an urgent need for basic molecular research to provide key answers regarding the molecular mechanism underlying olaparib function. In this study, we evaluated how treatment with metformin would affect the anti-tumor efficacy of olaparib and whether it was possible to achieve synthetic lethality in wild-type BRCA ovarian cancer cells [39,40]. The results demonstrate that olaparib 20 µM-metformin 20 mM combination treatment for 24 h significantly decreased the viability of SKOV-3 cells to 56% and that of OV-90 cells to 80%. Cell lines with a mutated p53 gene (OV-90) might be more sensitive to therapy if exposed to compounds for a prolonged period (Figure 1D,E). The “BRCAness” phenotype may be the result of homologous recombination deficiency due to dysfunction of genes other than BRCA1/2, such as TP53. The high frequency of deletions and insertions in the TP53 gene observed in these tumors may be the result of a deficient DNA-repair pathway, which makes the tumor more sensitive to cell death [41]. It is difficult to determine the percentage of ovarian cancer cases associated with disturbances in the HR process (resulting from mutations in genes other than BRCA 1/2). Disturbances in expression, regulation, or mutation of the HR genes are observed in almost half of the patients with advanced ovarian cancer. Although they mainly affect the BRCA1 and BRCA2 genes, they have been detected in other genes such as RAD51C hypermethylation (RAD51 paralog C), ATM (ataxia telangiectasia mutated serine-protein kinase gene), or ATR mutations, and mutations of the HR interacting Fanconi anemia gene [42,43,44].

Cancer cells have a higher output level of ROS than healthy cells, which makes them susceptible to changes in the intracellular antioxidant systems responsible for maintaining redox balance [35]. Unrepaired single-strand breaks (SSBs) resulting from oxidative damage are directed to HR and contribute significantly to PARPi toxicity. PARP1 is involved in the regulation of mitochondrial function and oxidative metabolism [36,45]. Thus, the repair of oxidative damage may contribute to changes in the toxicity of PARPis [46,47]. In this study, both drugs induced ROS production, and it was one of the earliest signs of the activity of the tested compounds in EOC. Metformin can oppose the Warburg effect in favor of oxidative phosphorylation as a result of 5’ AMP-activated protein kinase (AMPK) activation, and thus acts as a metabolic tumor suppressor [48]. This biguanide derivative is a partial inhibitor of complex 1 of the mitochondrial electron transport chain. Metformin increases the level of ROS in OVCAR3, CAOV3, and SKOV3 ovarian cancer cells [13]. An AMPK-independent upstream pathway for metformin can lead to the genesis of ROS and damage to the mitochondria in cancer cells and thus lead to their death [49,50]. The inhibition of PARP activity by olaparib increases the levels of NADPH oxidases 1 and 4, leading to ROS production [36]. Lahiguera et al. showed that incubation with metformin increases mitochondrial ROS to a greater level in Trp53/BRCA2-deleted cells than in Trp53-deleted cells [51].

P53, a key tumor-suppressor protein involved in apoptosis, DNA repair, cell cycle, and senescence, is encoded by the TP53 gene, which is the most frequently mutated gene in tumors [52,53]. In response to different forms of cellular stress, p53 activates the DNA damage response and causes cell-cycle arrest. Different protein–protein interactions and posttranslational modifications regulate its functions and stability [54,55]. High-grade serous carcinomas often involve p53 mutations [56] and such mutations are associated with tumor progression, metastasis, adverse clinical outcome, and the development of chemoresistance in ovarian cancer [57]. Mutations in p53 can be categorized as gain of function, which are caused by single amino acid alterations, typically in the DNA binding domain, or loss of function (p53 null) [58]. The OV-90 cell line carries a S215R p53 mutation, whereas SKOV-3 lacks a TP53 functional gene because of a single nucleotide deletion at position 267 (codon 90) [30,59,60,61]. The present results indicate that the combination of metformin and olaparib may significantly downregulate p53 expression in p53^MUT^ ovarian cancer cells. Consistent with the studies by Park et al. and Patel et al., SKOV-3 cells did not show p53 expression [61,62,63]. Tumor-associated p53 mutations may also alter metabolic pathways including glycolysis and stimulate the Warburg effect [64]. Several studies have shown that p53 is involved in the anti-tumor activity of metformin. At high doses, metformin inhibits the mitochondrial respiratory chain complex I and activates AMPK, which can further induce p53 phosphorylation and activation in melanoma [65]. Metformin inhibits MdmX (Mdm4, a p53 binding protein with structural similarity to Mdm2) expression, leading to p53 activation and cell death [66]. Thus, metformin inhibits cancer cell growth and survival in both p53-dependent and p53-independent ways. Smeby et al. showed that talazoparib, another PARPi, increases the number of TP53-positive nuclei in p53^WT^ cells, suggesting that the response to PARPis may provide a link between wild-type TP53 and HR deficiency, connected with RAD51 [67]. However, long-term (five days) treatment with 5 μM olaparib downregulates p53 in the p53^MUT^ ovarian cancer cell line OVCAR-3, whereas in the p53^WT^ cell line A2780, it upregulates p53 expression [68]. Ishiguro et al. reported p53 accumulation after triapine treatment in OV-90 cells, and suggested that this particular cell line may contain both wild-type and mutated TP53 genes [63].

The inhibition of mitochondrial complex I by metformin induces metabolic stress [69]. On the other hand, PARPis induce DNA replication stress via nucleotide exhaustion and PARP trapping [70]. The simultaneous occurrence of both types of cellular stress may transmit signals to the mitochondria, as suggested by the decreased mitochondrial membrane potential due to increased ROS production (Figure 3B). Such mitochondrial stress is triggered by DNA damage, which also induces apoptosis by phosphorylating mitochondrial proteins involved in the release of cytochrome c [71]. Metformin combined with 2.5 mM glucose upregulates cytosolic cytochrome c in SKOV3 cells and decreases mitochondrial membrane potential, suggesting that low glucose and metformin trigger cell apoptosis through the mitochondria-associated pathway [38]. Metformin (20 mM) and fingolimod (20 μM) synergistically affect the levels of cytochome c and loss of mitochondrial membrane potential [72]. Metformin induces apoptosis by upregulating cytochrome c expression in SKOV-3 cells; however, cell death is enhanced by the combination of carboplatin and/or paclitaxel [73]. Combined inhibition of PARP by olaparib and XIAP (X-linked inhibitor of apoptosis protein) by embelin synergistically cause the release of cytochrome c from mitochondria into the cytosol in breast cancer cells [74]. Another study indicated that olaparib treatment only slightly increases basal cytochrome c release in thyroid carcinoma [75]. In this study, olaparib combined with metformin increased ROS production, probably leading to mitochondrial damage and ultimately abolishing the protective effect of PARPi on mitochondria. Previously, it was confirmed that PARP1 regulates Pol γ repair activity in a NAD-dependent manner in mitochondrial base excision repair, and olaparib increases mtDNA mutations [76]. A similar study showed that the PARPi PJ34 protects mitochondria, but also induces DNA-damage mediated apoptosis in combination with cisplatin or temozolomide [77].

Gene mutations confer cancer cells immortality and thus resistance to programmed cell death, thereby achieving the ability of unlimited proliferation [78]. We showed that metformin and olaparib could induce apoptosis. The present results contribute to a better understanding of the molecular basis of the apoptotic properties of the investigated drugs in ovarian cancer cells. Metformin may promote apoptosis in A2780, ES2, and SKOV-3 ovarian cancer cell lines [79]. Similar results were reported in other epithelial ovarian cancer cell lines, such as PA-1 and OVCAR-3 [80]. However, a study reported that apoptosis of SKOV-3, OVCAR-3, and HO8910 ovarian cancer cell lines was weakly induced by metformin alone and only under low glucose cell culture conditions [38]. Cell death of lymphocyte-derived cell lines induced by olaparib varies between cell types; Reh and Granta-519 cells die by apoptosis, whereas U698 and JVM-2 cells undergo necrosis [81]. PARPis inhibit cell proliferation and promote apoptosis in the human breast cancer cell line Bcap37, and this effect is enhanced by paclitaxel [82].

In this study, detection of DNA fragmentation during apoptosis, as well as measurement of caspase 3/7 activation, clearly revealed that combination therapy resulted in a higher level of apoptosis hallmarks than monotherapy in both investigated cell lines. However, SKOV-3 cells were more susceptible to apoptosis induced by the combination of the drugs than OV-90 cells. These observations are consistent with previous studies. Olaparib increases the metformin-induced activation of AMPK. Metformin at 20 mM increases the level of cleaved caspase-3 in SKOV-3 and Hey cell lines [83]. Olaparib at 5 μM slightly increased the cleavage of caspase-3 in high-grade serous ovarian carcinoma cells [84]. However, reports also show that the combination of metformin and olaparib does not induce marked apoptosis in BRCA1-intact ovarian cancer cells, although cell-cycle analysis revealed a significant S-phase arrest. Combination therapy also significantly inhibited SKOV3-generated ovarian tumor xenografts [85]. The cell death mechanisms induced by metformin and olaparib are pleiotropic and depend on the type of cell line. The sensitivity of tumors to olaparib depends on the metabolic profile. Thus, by increasing the glycolysis rate, metformin may alter the capacity of olaparib to induce apoptosis [51].

The effectiveness of the metformin and olaparib combination was confirmed in other studies. This treatment strategy is currently being investigated in the ENDOLA phase I/II study [86]. Data show that metformin can reverse PARPi-induced epithelial-mesenchymal transition and PD-L1 upregulation in triple-negative breast cancer by sensitizing PARPi-resistant cells to cytotoxic T cells [87]. Another study demonstrated that biguanides in combination with PARPis synergistically reduce epithelial–mesenchymal transition, proliferation, and survival of ovarian drug-resistant cancer cells [78]. Biguanides alone (phenformin, metformin) or in combination with low doses of olaparib inhibit the survival of A2780PAR cells and their resistant clone, A2780CR [78]. Despite the emergence of promising new clinical trials, a large group of patients with ovarian cancer is still excluded. This study provides a basis for further preclinical tests expanding the target group to patients with BRCA^WT^ ovarian cancer.

## 4. Materials and Methods

### 4.1. Reagents

Culture media (RPMI 1640, cat. 72400054 and DMEM, cat. 32430100) were obtained from Gibco (Thermo Fisher Scientific, Waltham, MA, USA). Fetal bovine serum (FBS) was from Capricorn Scientific GmbH (Ebsdorfergrund, Germany). Trypsin-EDTA, penicillin, streptomycin, metformin, MTT, Hoechst 33258, PI and JC-1 were from Sigma-Aldrich (St. Louis, MO, USA). Apo-ONE^®^ Homogeneous Caspase 3/7 Assay was from Promega Corporation (Madison, WI, USA). PARPi (AZD2281; O) was purchased from Selleckchem. Apo-BrdU In Situ DNA Fragmentation Assay Kit and Annexin V-FITC were supplied by BioVision Inc. (Milpitas, CA, USA). Other chemicals and solvents were of high analytical grade and were obtained from Sigma-Aldrich or Avantor Performance Materials Poland S.A. (Gliwice, Poland).

### 4.2. Cell Culture and Drug Administration

OV-90 [human malignant papillary serous carcinoma, American Type Culture Collection (ATCC) CRL-11732™], SKOV-3 (human ovarian adenocarcinoma, ATCC HTB-77) cell lines were purchased from the ATCC (Rockville, MD, USA). During this study, cells were thawed and passaged within two months in each experiment. SKOV-3 and OV-90 cells were cultured in RPMI 1640 (11.11 mM D-glucose) and DMEM (25 mM D-glucose), respectively. Both media were supplemented with 10% FBS, penicillin (10 U/mL) and streptomycin (50 µg/mL). Investigated cell lines were regularly checked for mycoplasma contamination, as described previously [88]. The cells were cultured under an atmosphere of 5% CO_2_ and 95% air at 37 °C.

### 4.3. MTT Assay

Drug cytotoxicity against SKOV-3 and OV-90 ovarian cancer cells was estimated using the standard microplate MTT colorimetric method. Logarithmically growing cells (1 × 104 SKOV-3 and OV-90 cells/well) were seeded on 96-well plates, and 24 h later were treated with different concentrations (10–80 µM) of PARPi (AZD2281) and metformin (10–80 mM) and incubated for another 24–72 h. At the end of the incubation period, the medium was removed and 50 µL of MTT (at a final concentration of 0.5 mg/mL) was added to each well after two washes with phosphate buffered saline (PBS). Subsequently, microplates were incubated for 4 h. Then the violet formazan crystals were dissolved in 100 µL DMSO per well. Absorbance was measured at 570 nm with a microplate reader (Awareness Technology Inc., Palm City, FL, USA) [89].

To select the most effective ratio of olaparib: metformin, several concentration ratios of the compounds (1:1000) were tested. To analyze the drug interactions between olaparib combined with metformin, the CDI was calculated [90]. CDI is defined by the following formula: CDI = AB/(A × B). According to the absorbance of each group, AB is the ratio of the two-drug combination group to the untreated control group, and A or B is the ratio of the single-drug group to the control group. CDI < 1 indicates synergism, CDI < 0.7 significant synergism, CDI = 1 additivity, and CDI >1 antagonism.

### 4.4. Clonogenic Assay

The effect of olaparib and metformin on cell growth was assessed using a clonogenic assay. For this analysis, 200 cells were plated onto 6-well plates in complete growth medium, and after overnight attachment, the cells were exposed to the indicated compounds for 24 h. The cells were then washed with medium and allowed to grow for 10–14 days under drug-free conditions. After that, the cell colonies were fixed with a methanol/acetic acid (7:1) solution for 10 min and stained with 0.5% crystal violet for 20 min. The plates were rinsed with water, air-dried, photographed, and evaluated for colony estimation. Colonies containing more than 50 cells were counted as described previously [91]. For each sample, the results from three replicates were averaged. To analyze the drug interaction between olaparib and metformin, the CDI was calculated [90].

### 4.5. Measurement of ROS Production

Production of ROS was measured using the 2′,7′-dichloro - dihydrofluorescein diacetate (DCFH2-DA) probe as described previously [91]. Intracellular ROS levels were determined directly in cell monolayers in 96-well microplates using a Fluoroskan Ascent FL microplate reader (Labsystems, Stockholm, Sweden). Cells were preincubated with DCFH2-DA in the culture medium at a final concentration of 5 µM for 30 min at 37 °C [20]. The kinetics of ROS generation in OV-90 and SKOV-3 cells after treatment with 20 µM olaparib and/or 20 mM metformin were measured for up to 90 min in the presence or absence of an antioxidant (1 mM NAC, N-acetylcysteine). In our experiments, we used 400 µM of H2O2 as a positive control (data not shown). The fluorescence of DCF was measured at 530 nm after excitation at 485 nm (DCFH2-DA, after deacetylation to DCFH2, is oxidized intracellularly to its fluorescent derivative, DCF). Assays were performed in modified Hank’s buffered salt solution (HBSS; 140 mM NaCl, 5 mM KCl, 0.8 mM MgCl_2_, 1.8 mM CaCl_2_, 1 mM Na_2_HPO_4_, 10 mM HEPES. and 1% glucose, pH 7.0, without phenol red).

### 4.6. Mitochondrial Membrane Potential (ΔΨm)

Cells were seeded into 96-well microplates. After 24 h, cells were incubated with the drugs or CCCP, an uncoupling mitochondrial agent (10 µM) for 24 h. In the experiments with the antioxidant the cells were preincubated with 1 mM NAC for 1 h and then treated with drugs for 24 h. Additional positive controls were cells treated with 5 µM CPT for 24 h. At the end of treatment, the medium was removed, and the cells were incubated in total darkness with 5 µM JC-1 in HBSS for 30 min at 37 °C. The fluorescence of both JC-1 monomers and dimers was measured on a Fluoroskan Ascent FL microplate reader using filter pairs of 530/590 nm (dimers) and 485/538 nm (monomers). Prior to fluorescence measurements and photography, cells were washed twice with HBSS to remove the dye, which otherwise could have adsorbed on the microplate well plastic and distorted measurements. The results shown in the figures are expressed as a ratio of dimer to monomer fluorescence in relation to the control fluorescence ratio, taken as 100%. The cells presented in the images were incubated with drugs for 48 h. JC-1 fluorescence was photographed immediately after drug treatment with an inverted Olympus IX70 fluorescence microscope (Olympus, Tokyo, Japan).

### 4.7. Morphological Assessment of Apoptosis and Necrosis—Double staining with Hoechst 3325 and Propidium Iodide and Double Staining with Orange Acridine and Ethidium Bromide

To determine the ratio between live, apoptotic, and necrotic cell fractions, simultaneous cell staining with Hoechst 33258 and PI was performed as described previously [92]. These fluorescent dyes vary in their spectral characteristics and ability to penetrate cells. The analysis was performed with the Olympus IX70 fluorescence microscope. Cells were cultured with the drugs for 24 h. Then, the cells were removed from the culture dishes by trypsinization, centrifuged, and suspended in PBS to a final concentration of 1 × 10^6^ cells/mL. One microliter of Hoechst 33258 (0.13 mM) and 1 µL of PI (0.23 mM) were added to 100 µL of the cell suspension. At least 100 cells were counted under a microscope on each slide and each experiment was done in triplicate. Cells were classified as live, apoptotic or necrotic on the basis of their morphological and staining characteristics and the percentages of particular cell types were determined from the total number of cells. Cells were classified as live (bright blue fluorescence), early apoptotic cells (cells showing intensive blue fluorescence), late apoptotic cells (blue–violet stained cells with concomitant apoptotic morphology), and necrotic cells (red fluorescence). Additional positive controls were cells treated with 5 µM CPT, for 24 h.

### 4.8. Caspase 3/7 Assay

The activity of caspase-3 and -7 was estimated with the Apo-ONE^®^ Homogeneous Caspase 3/7 Assay (Promega Corporation, Madison, WI, USA) according to the manufacturer’s protocols, as described in our previous article [93]. Measurement of caspase activation in the control and treated cells seeded in 96 black well plates was recorded by monitoring changes in fluorescence after 24 h of incubation with the investigated compounds. The intensity of fluorescence was measured using a Fluoroskan Ascent FL plate reader (Labsystems, Helsinki, Finland). Cysteine protease activity was expressed as the ratio of fluorescence of the treated samples relative to the corresponding untreated controls, the latter taken as 100%. Z-FA-FMK (R&D Systems, Minneapolis, MN, USA), a caspase-3 inhibitor, was used in the control experiments to confirm that the observed fluorescence in both the control and the drug-treated cells was due to the presence of caspases-3/7 in the samples. As additional positive controls, cells treated with 5 µM CPT for 24 h were used to independently induce apoptosis and confirm that the observed fluorescence was due to the presence of cysteine proteases in the samples.

### 4.9. Measurements of DNA Damage during Apoptosis—TUNEL Assay

The Apo-BrdU DNA Fragmentation Assay Kit (BioVision, Milpitas, California, USA) was used to examine DNA damage during apoptosis according to the protocol described in our previous articles [94,95]. This method enables the detection of the early stage of apoptosis by labeling the 3′ OH ends of single- and double-stranded DNA fragments with Br-dUTP (bromolated deoxyuridine triphosphate nucleotides). The Br-dUTP fragments are detected by the fluorescein labeled anti-BrdU monoclonal antibody, which produces a brighter signal. The control and drug-treated cells were fixed in 4% paraformaldehyde freshly prepared in PBS and incubated for 1 h at 37 °C in DNA Labeling Solution containing a terminal deoxynucleotidyl transferase (TdT) Reaction Buffer, Tdt, and Br-dUTP. Next, the cells were resuspended in an antibody solution containing an anti-BrdU-FITC antibody (in total darkness for 30 min at room temperature) and incubated with the propidium iodide/RNase A solution. The cell fluorescence was measured by flow-cytometry (LSR II, Becton Dickinson). The green fluorescence of FITC at 520 nm and the red fluorescence of propidium iodide at 623 nm were detected. The number of TUNEL-positive cells was expressed as a percentage of the total number of cells in the sample. As additional, positive controls, cells treated with 5 µM CPT for 24 h were used to independently induce apoptosis (data not shown).

### 4.10. Phosphatidylserine Externalization Measurement

After 24 h of treatment with olaparib or metformin alone and in combination, SKOV-3 and OV-90 cells were collected from 60 mm Petri dishes, centrifuged (200× *g*, 5 min), and washed with HBSS. Control and drug-treated cells (5 × 105) were resuspended in 500 μL binding buffer containing 5 μL of Annexin V FITC and stained for 15 min at room temperature. Next, control and treated cells were transferred (100 μL, 1 × 105 cells/well) to 96-well black plates, centrifuged to remove unbound Annexin V (200× *g*, 5 min), and resuspended in 100 μL fresh binding buffer. Then, the fluorescence intensity was analyzed on a Fluoroskan Ascent FL microplate reader (Labsystems) using 485 nm excitation and 525 nm emission wavelengths.

### 4.11. Western Blot Analysis

The cells were treated and lysed in cell extraction buffer (Invitrogen™, Waltham, MA, USA) containing a protease inhibitor cocktail and PMSF (Sigma-Aldrich) in accordance with the manufacturer’s protocol. The protein concentration was determined using the Bradford method. Proteins (35 µg per lane) were separated by SDS polyacrylamide gel electrophoresis and transferred onto 0.45 µm PVDF membranes using semi-dry transfer with the Trans-Blot Turbo Transfer System (Bio-Rad, Hercules, CA, USA). After blocking nonspecific sites with 5% non-fat dry milk, membranes were incubated with rabbit monoclonal antibody at a dilution of 1/1000 against p53 (cat. # 2527) and cytochrome c (cat. # 11940) from Cell Signaling Technology, Inc. (Danvers, MA, USA), and mouse monoclonal anti-β-actin antibody (cat. A1878, Sigma-Aldrich). Membranes were then exposed to anti-rabbit IgG horseradish peroxidase-conjugated secondary antibody (cat. #7074, Cell Signaling Technology) or anti-mouse IgG HRP-conjugated secondary antibody (# A28177, Invitrogen, Thermo Fisher Scientific, Waltham, MA, USA), followed by detection using a chemiluminescent substrate (Thermo Fisher Scientific, Waltham, MA, USA). Immunoreactive bands were visualized using a DNR MicroChemi system. Band intensities were quantified using ImageJ software (NIH, Bethesda, MD, USA). The integrated optical density of the bands was measured in a digitized image. Relative protein levels were expressed as the ratio of the densitometric volume of the test band to that of the respective β-actin band. CPT treatment at 5 µM for 24 h was used as a positive control.

### 4.12. Statistical Analysis

The data are presented as the mean ± SD of at least three independent experiments. Statistical analyses were performed with Student’s *t*-test and ANOVA with Tukey’s post hoc test for multiple comparisons as appropriate (StatSoft, Tulsa, OK, USA) [94]. P values of less than 0.05 were considered as statistically significant.

## 5. Conclusions

This study demonstrated that the combination of metformin with clinically available targeted drugs, such as olaparib, have a strong cytotoxic effect in ovarian cancer cells. The proliferation and clonogenicity of wild-type ovarian cancer cells were pronouncedly suppressed by the combination of metformin and olaparib compared with the effect of each drug alone. Drugs combination suppresses cell survival, producing a high level of apoptosis hallmarks in EOC. The response to the combined action of metformin–olaparib involves the effect of ROS on damaging cellular components, such as mitochondria, thereby leading to cell death. Moreover, increased ROS production might be the key molecular mechanisms by which metformin sensitizes wild-type BRCA EOC cells to olaparib. The combination of olaparib with metformin may indicate a synergistic effect, especially after prolonged drug action in both tested cell models with p53^MUT^ and p53 null. Further investigation into the effectiveness of this combination in vivo may lead to widespread treatment opportunities for ovarian cancer patients in the future.

## Data Availability

The datasets presented during in the current study are available from the corresponding author on reasonable request.

## Supplementary Materials

The following are available online at https://www.mdpi.com/article/10.3390/ijms221910557/s1.

## References

[1] Momenimovahed Z., Tiznobaik A., Taheri S., Salehiniya H. (2019). Ovarian cancer in the world: Epidemiology and risk factors. Int. J. Womens Health.

[2] Budiana I.N.G., Angelina M., Pemayun T.G.A. (2019). Ovarian cancer: Pathogenesis and current recommendations for prophylactic surgery. J. Turk. Ger. Gynecol. Assoc..

[3] McGee J., Peart T.M., Foley N., Bertrand M., Prefontaine M., Sugimoto A., Ettler H., Welch S., Panabaker K. (2019). Direct Genetics Referral Pathway for High-Grade Serous Ovarian Cancer Patients: The “Opt-Out” Process. J. Oncol..

[4] Farmer H., McCabe N., Lord C.J., Tutt A.N., Johnson D.A., Richardson T.B., Santarosa M., Dillon K.J., Hickson I., Knights C. (2005). Targeting the DNA repair defect in BRCA mutant cells as a therapeutic strategy. Nature.

[5] The Cancer Genome Atlas Research Network (2011). Integrated genomic analyses of ovarian carcinoma. Nature.

[6] Buttarelli M., De Donato M., Raspaglio G., Babini G., Ciucci A., Martinelli E., Baccaro P., Pasciuto T., Fagotti A., Scambia G. (2020). Clinical Value of lncRNA MEG3 in High-Grade Serous Ovarian Cancer. Cancers.

[7] van Zyl B., Tang D., Bowden N.A. (2018). Biomarkers of platinum resistance in ovarian cancer: What can we use to improve treatment. Endocrine.-Relat. Cancer.

[8] Chen K., Li Y., Guo Z., Zeng Y., Zhang W., Wang H. (2020). Metformin: Current clinical applications in nondiabetic patients with cancer. Aging.

[9] Cioce M., Pulito C., Strano S., Blandino G., Fazio V.M. (2020). Metformin: Metabolic Rewiring Faces Tumor Heterogeneity. Cells.

[10] Yin G., Liu Z., Wang Y., Sun L., Wang L., Yao B., Liu R., Chen T., Niu Y., Liu Q. (2019). ZNF503 accelerates aggressiveness of hepatocellular carcinoma cells by down-regulation of GATA3 expression and regulated by microRNA-495. Am. J. Transl. Res..

[11] El Shorbagy S., abuTaleb F., Labib H.A., Ebian H., Harb O.A., Mohammed M.S., Rashied H.A., Elbana K.A., Haggag R. (2020). Prognostic Significance of VEGF and HIF-1 α in Hepatocellular Carcinoma Patients Receiving Sorafenib Versus Metformin Sorafenib Combination. J. Gastrointest. Cancer.

[12] Orecchioni S., Reggiani F., Talarico G., Mancuso P., Calleri A., Gregato G., Labanca V., Noonan D.M., Dallaglio K., Albini A. (2015). The biguanides metformin and phenformin inhibit angiogenesis, local and metastatic growth of breast cancer by targeting both neoplastic and microenvironment cells. Int. J. Cancer.

[13] Petrushev B., Tomuleasa C., Soritau O., Aldea M., Pop T., Susman S., Kacso G., Berindan I., Irimie A., Cristea V. (2012). Metformin plus PIAF combination chemotherapy for hepatocellular carcinoma. Exp. Oncol..

[14] Markowska A., Leracz-Jacczak A. (2018). Metformin in cancer treatment. Curr. Gynecol. Oncol..

[15] Baloch T., López-Ozuna V.M., Wang Q., Matanis E., Kessous R., Kogan L., Yasmeen A., Gotlieb W.H. (2019). Sequential therapeutic targeting of ovarian Cancer harboring dysfunctional BRCA1. BMC Cancer.

[16] Ronson G.E., Piberger A.L., Higgs M.R., Olsen A.L., Stewart G.S., McHugh P.J., Petermann E., Lakin N.D. (2018). PARP1 and PARP2 stabilise replication forks at base excision repair intermediates through Fbh1-dependent Rad51 regulation. Nat. Commun..

[17] Pujade-Lauraine E., Ledermann J.A., Selle F., Gebski V., Penson R.T., Oza A.M., Korach J., Huzarski T., Poveda A., Pignata S. (2017). Olaparib tablets as maintenance therapy in patients with platinum-sensitive, relapsed ovarian cancer and a BRCA1/2 mutation (SOLO2/ENGOT-Ov21): A double-blind, randomised, placebo-controlled, phase 3 trial. Lancet Oncol..

[18] Mirza M.R., Monk B.J., Herrstedt J., Oza A.M., Mahner S., Redondo A., Fabbro M., Ledermann J.A., Lorusso D., Vergote I. (2016). Niraparib Maintenance Therapy in Platinum-Sensitive, Recurrent Ovarian Cancer. N. Engl. J. Med..

[19] Coleman R.L., Oza A.M., Lorusso D., Aghajanian C., Oaknin A., Dean A., Colombo N., Weberpals J.I., Clamp A., Scambia G. (2017). Rucaparib maintenance treatment for recurrent ovarian carcinoma after response to platinum therapy (ARIEL3): A randomised, double-blind, placebo-controlled, phase 3 trial. Lancet.

[20] Klotz D.M., Wimberger P. (2020). Overcoming PARP inhibitor resistance in ovarian cancer: What are the most promising strategies?. Arch. Gynecol. Obstet..

[21] Pellegrino B., Mateo J., Serra V., Balmaña J. (2019). Controversies in oncology: Are genomic tests quantifying homologous recombination repair deficiency (HRD) useful for treatment decision making?. ESMO Open.

[22] Ahmad A., Lin Z.P., Zhu Y.-L., Lo Y.-C., Moscarelli J., Xiong A., Korayem Y., Huang P.H., Giri S., LoRusso P. (2018). Combination of triapine, olaparib, and cediranib suppresses progression of BRCA-wild type and PARP inhibitor-resistant epithelial ovarian cancer. PLoS ONE.

[23] Randall M., Burgess K., Buckingham L., Usha L. (2020). Exceptional Response to Olaparib in a Patient With Recurrent Ovarian Cancer and an Entire BRCA1 Germline Gene Deletion. J. Natl. Compr. Cancer Netw..

[24] Carneiro B.A., Collier K.A., Nagy R.J., Pamarthy S., Sagar V., Fairclough S., Odegaard J., Lanman R.B., Costa R., Taxter T. (2018). Acquired Resistance to Poly (ADP-ribose) Polymerase Inhibitor Olaparib in BRCA2-Associated Prostate Cancer Resulting From Biallelic BRCA2 Reversion Mutations Restores Both Germline and Somatic Loss-of-Function Mutations. JCO Precis. Oncol..

[25] Mengwasser K.E., Adeyemi R.O., Leng Y., Choi M.Y., Clairmont C., D’Andrea A.D., Elledge S.J. (2019). Genetic Screens Reveal FEN1 and APEX2 as BRCA2 Synthetic Lethal Targets. Mol. Cell.

[26] Kaelin W.G. (2005). The Concept of Synthetic Lethality in the Context of Anticancer Therapy. Nat. Rev. Cancer.

[27] Gentric G., Kieffer Y., Mieulet V., Goundiam O., Bonneau C., Nemati F., Hurbain I., Raposo G., Popova T., Stern M.H. (2019). PML-Regulated Mitochondrial Metabolism Enhances Chemosensitivity in Human Ovarian Cancers. Cell Metab..

[28] Wilson A.J., Stubbs M., Liu P., Ruggeri B., Khabele D. (2018). The BET inhibitor INCB054329 reduces homologous recombination efficiency and augments PARP inhibitor activity in ovarian cancer. Gynecol. Oncol..

[29] Hurley R.M., Wahner Hendrickson A.E., Visscher D.W., Ansell P., Harrell M.I., Wagner J.M., Negron V., Goergen K.M., Maurer M.J., Oberg A.L. (2019). 53BP1 as a potential predictor of response in PARP inhibitor-treated homologous recombination-deficient ovarian cancer. Gynecol. Oncol..

[30] Domcke S., Sinha R., Levine D.A., Sander C., Schultz N. (2013). Evaluating cell lines as tumour models by comparison of genomic profiles. Nat. Commun..

[31] Sanij E., Hannan K.M., Xuan J., Yan S., Ahern J.E., Trigos A.S., Brajanovski N., Son J., Chan K.T., Kondrashova O. (2020). CX-5461 activates the DNA damage response and demonstrates therapeutic efficacy in high-grade serous ovarian cancer. Nat. Commun..

[32] Loo D.T. (2002). TUNEL assay. An overview of techniques. Methods Mol. Biol..

[33] Hjortkjær M., Malik Aagaard Jørgensen M., Waldstrøm M., Ørnskov D., Søgaard-Andersen E., Jakobsen A., Dahl-Steffensen K. (2019). The clinical importance of BRCAness in a population-based cohort of Danish epithelial ovarian cancer. Int. J. Gynecol. Cancer.

[34] Pizzo S.V., Dahmani Z., Addou-Klouche L., Gizard F., Dahou S., Messaoud A., Chahinez Djebri N., Benaissti M.I., Mostefaoui M., Terbeche H. (2020). Metformin partially reverses the inhibitory effect of co-culture with ER-/PR-/HER2+ breast cancer cells on biomarkers of monocyte antitumor activity. PLoS ONE.

[35] Peuget S., Bonacci T., Soubeyran P., Iovanna J., Dusetti N.J. (2014). Oxidative stress-induced p53 activity is enhanced by a redox-sensitive TP53INP1 SUMOylation. Cell Death Differ..

[36] Hou D., Liu Z., Xu X., Liu Q., Zhang X., Kong B., Wei J.-J., Gong Y., Shao C. (2018). Increased oxidative stress mediates the antitumor effect of PARP inhibition in ovarian cancer. Redox Biol..

[37] Alhourani A.H., Tidwell T.R., Bokil A.A., Rosland G.V., Tronstad K.J., Soreide K., Hagland H.R. (2021). Metformin treatment response is dependent on glucose growth conditions and metabolic phenotype in colorectal cancer cells. Sci. Rep..

[38] Ma L., Wei J., Wan J., Wang W., Wang L., Yuan Y., Yang Z., Liu X., Ming L. (2019). Low glucose and metformin-induced apoptosis of human ovarian cancer cells is connected to ASK1 via mitochondrial and endoplasmic reticulum stress-associated pathways. J. Exp. Clin. Cancer Res..

[39] Guo E., Ishii Y., Mueller J., Srivatsan A., Gahman T., Putnam C.D., Wang J.Y.J., Kolodner R.D. (2020). FEN1 endonuclease as a therapeutic target for human cancers with defects in homologous recombination. Proc. Natl. Acad. Sci. USA.

[40] Beaufort C.M., Helmijr J.C., Piskorz A.M., Hoogstraat M., Ruigrok-Ritstier K., Besselink N., Murtaza M., van I.W.F., Heine A.A., Smid M. (2014). Ovarian cancer cell line panel (OCCP): Clinical importance of in vitro morphological subtypes. PLoS ONE.

[41] Silwal-Pandit L., Langerod A., Borresen-Dale A.L. (2017). TP53 Mutations in Breast and Ovarian Cancer. Cold Spring Harb. Perspect. Med..

[42] Angeli D., Salvi S., Tedaldi G. (2020). Genetic Predisposition to Breast and Ovarian Cancers: How Many and Which Genes to Test?. Int. J. Mol. Sci..

[43] Cantor S.B., Guillemette S. (2011). Hereditary breast cancer and the BRCA1-associated FANCJ/BACH1/BRIP1. Future Oncol..

[44] Niraj J., Farkkila A., D’Andrea A.D. (2019). The Fanconi Anemia Pathway in Cancer. Annu. Rev. Cancer Biol..

[45] Bai P., Cantó C., Oudart H., Brunyánszki A., Cen Y., Thomas C., Yamamoto H., Huber A., Kiss B., Houtkooper R.H. (2011). PARP-1 Inhibition Increases Mitochondrial Metabolism through SIRT1 Activation. Cell Metab..

[46] Verhagen C.V.M., de Haan R., Hageman F., Oostendorp T.P.D., Carli A.L.E., O’Connor M.J., Jonkers J., Verheij M., van den Brekel M.W., Vens C. (2015). Extent of radiosensitization by the PARP inhibitor olaparib depends on its dose, the radiation dose and the integrity of the homologous recombination pathway of tumor cells. Radiother. Oncol..

[47] Giovannini S., Weller M.-C., Repmann S., Moch H., Jiricny J. (2019). Synthetic lethality between BRCA1 deficiency and poly(ADP-ribose) polymerase inhibition is modulated by processing of endogenous oxidative DNA damage. Nucleic Acids Res..

[48] Daugan M., Wojcicki A.D., d’Hayer B., Boudy V. (2016). Metformin: An anti-diabetic drug to fight cancer. Pharmacol. Res..

[49] Chan D.K., Miskimins W. (2012). Metformin and phenethyl isothiocyanate combined treatment in vitro is cytotoxic to ovarian cancer cultures. J. Ovarian Res..

[50] Ohnishi S., Mizutani H., Kawanishi S. (2016). The enhancement of oxidative DNA damage by anti-diabetic metformin, buformin, and phenformin, via nitrogen-centered radicals. Free Radic. Res..

[51] Lahiguera Á., Hyroššová P., Figueras A., Garzón D., Moreno R., Soto-Cerrato V., McNeish I., Serra V., Lazaro C., Barretina P. (2020). Tumors defective in homologous recombination rely on oxidative metabolism: Relevance to treatments with PARP inhibitors. EMBO Mol. Med..

[52] Vousden K.H., Ryan K.M. (2009). p53 and metabolism. Nat. Rev. Cancer.

[53] Goh A.M., Coffill C.R., Lane D.P. (2011). The role of mutant p53 in human cancer. J. Pathol..

[54] Gu B., Zhu W.-G. (2012). Surf the Post-translational Modification Network of p53 Regulation. Int. J. Biol. Sci..

[55] Vousden K.H., Prives C. (2009). Blinded by the Light: The Growing Complexity of p53. Cell.

[56] Rosen D.G., Yang G., Liu G., Mercado-Uribe I., Chang B., Xiao X.S., Zheng J., Xue F.X., Liu J. (2009). Ovarian cancer: Pathology, biology, and disease models. Front. Biosci..

[57] Moll U.M., Wolff S., Speidel D., Deppert W. (2005). Transcription-independent pro-apoptotic functions of p53. Current Opinion in Cell Biol..

[58] Meng X., Bi J., Li Y., Yang S., Zhang Y., Li M., Liu H., Li Y., McDonald M., Thiel K. (2018). AZD1775 Increases Sensitivity to Olaparib and Gemcitabine in Cancer Cells with p53 Mutations. Cancers.

[59] Langland G.T., Yannone S.M., Langland R.A., Nakao A., Guan Y., Long S.B., Vonguyen L., Chen D.J., Gray J.W., Chen F. (2010). Radiosensitivity profiles from a panel of ovarian cancer cell lines exhibiting genetic alterations in p53 and disparate DNA-dependent protein kinase activities. Oncol. Rep..

[60] Zhang Z., Alaee M., Danesh G., Pasdar M. (2016). Plakoglobin Reduces the in vitro Growth, Migration and Invasion of Ovarian Cancer Cells Expressing N-Cadherin and Mutant p53. PLoS ONE.

[61] Patel S., Singh N., Kumar L. (2016). Anticancer role of antidiabetic drug Metformin in ovarian cancer cells. Int. J. Cancer Ther. Oncol..

[62] Park E.Y., Woo Y., Kim S.J., Kim D.H., Lee E.K., De U., Kim K.S., Lee J., Jung J.H., Ha K.-T. (2016). Anticancer Effects of a New SIRT Inhibitor, MHY2256, against Human Breast Cancer MCF-7 Cells via Regulation of MDM2-p53 Binding. Int. J. Biol. Sci..

[63] Ishiguro K., Zhu Y.L., Lin Z.P., Penketh P.G., Shyam K., Zhu R., Baumann R.P., Sartorelli A.C., Rutherford T.J., Ratner E.S. (2016). Cataloging antineoplastic agents according to their effectiveness against platinum-resistant and platinum-sensitive ovarian carcinoma cell lines. J. Transl. Sci..

[64] Zhang C., Liu J., Liang Y., Wu R., Zhao Y., Hong X., Lin M., Yu H., Liu L., Levine A.J. (2013). Tumour-associated mutant p53 drives the Warburg effect. Nat. Commun..

[65] Cerezo M., Tichet M., Abbe P., Ohanna M., Lehraiki A., Rouaud F., Allegra M., Giacchero D., Bahadoran P., Bertolotto C. (2013). Metformin Blocks Melanoma Invasion and Metastasis Development in AMPK/p53-Dependent Manner. Mol. Cancer Ther..

[66] Gu J.J., Zhang Q., Mavis C., Czuczman M.S., Hernandez-Ilizaliturri F.J. (2015). Metformin Induces p53-Dependent Mitochondrial Stress in Therapy-Sensitive and -Resistant Lymphoma Pre-Clinical Model and Primary Patients Sample with B-Cell Non-Hodgkin Lymphoma (NHL). Blood.

[67] Smeby J., Kryeziu K., Berg K.C.G., Eilertsen I.A., Eide P.W., Johannessen B., Guren M.G., Nesbakken A., Bruun J., Lothe R.A. (2020). Molecular correlates of sensitivity to PARP inhibition beyond homologous recombination deficiency in pre-clinical models of colorectal cancer point to wild-type TP53 activity. EBioMedicine.

[68] Wang Z., Gao J., Zhou J., Liu H., Xu C. (2019). Olaparib induced senescence under P16 or P53 dependent manner in ovarian cancer. J. Gynecol. Oncol..

[69] Saini N., Yang X. (2018). Metformin as an anti-cancer agent: Actions and mechanisms targeting cancer stem cells. Acta Biochim. Biophys. Sin..

[70] Parsels L.A., Karnak D., Parsels J.D., Zhang Q., Velez-Padilla J., Reichert Z.R., Wahl D.R., Maybaum J., O’Connor M.J., Lawrence T.S. (2018). PARP1 Trapping and DNA Replication Stress Enhance Radiosensitization with Combined WEE1 and PARP Inhibitors. Mol. Cancer Res..

[71] Valdez B.C., Li Y., Murray D., Liu Y., Nieto Y., Champlin R.E., Andersson B.S. (2017). The PARP inhibitor olaparib enhances the cytotoxicity of combined gemcitabine, busulfan and melphalan in lymphoma cells. Leuk Lymphoma.

[72] Zhao Y., Zhang E., Lv N., Ma L., Yao S., Yan M., Zi F., Deng G., Liu X., He J. (2018). Metformin and FTY720 Synergistically Induce Apoptosis in Multiple Myeloma Cells. Cell. Physiol. Biochem..

[73] Patel S., Kumar L., Singh N. (2015). Metformin and epithelial ovarian cancer therapeutics. Cell. Oncol..

[74] Siraj A.K., Pratheeshkumar P., Parvathareddy S.K., Divya S.P., Al-Dayel F., Tulbah A., Ajarim D., Al-Kuraya K.S. (2018). Overexpression of PARP is an independent prognostic marker for poor survival in Middle Eastern breast cancer and its inhibition can be enhanced with embelin co-treatment. Oncotarget.

[75] Passaro C., Volpe M., Botta G., Scamardella E., Perruolo G., Gillespie D., Libertini S., Portella G. (2015). PARP inhibitor olaparib increases the oncolytic activity of dl922-947 in in vitro and in vivo model of anaplastic thyroid carcinoma. Mol. Oncol..

[76] Herrmann G.K., Russell W.K., Garg N.J., Yin Y.W. (2021). Poly(ADP-ribose) polymerase 1 regulates mitochondrial DNA repair in an NAD-dependent manner. J. Biol. Chem..

[77] Cseh A.M., Fabian Z., Quintana-Cabrera R., Szabo A., Eros K., Soriano M.E., Gallyas F., Scorrano L., Sumegi B. (2019). PARP Inhibitor PJ34 Protects Mitochondria and Induces DNA-Damage Mediated Apoptosis in Combination With Cisplatin or Temozolomide in B16F10 Melanoma Cells. Front. Physiol..

[78] Wang Q., López-Ozuna V.M., Baloch T., Bithras J., Amin O., Kessous R., Kogan L., Laskov I., Yasmeen A. (2019). Biguanides in combination with olaparib limits tumorigenesis of drug-resistant ovarian cancer cells through inhibition of Snail. Cancer Med..

[79] Tang G., Guo J., Zhu Y., Huang Z., Liu T., Cai J., Yu L., Wang Z. (2018). Metformin inhibits ovarian cancer via decreasing H3K27 trimethylation. Int. J. Oncol..

[80] Moon H.-s., Kim B., Gwak H., Suh D.H., Song Y.S. (2016). Autophagy and protein kinase RNA-like endoplasmic reticulum kinase (PERK)/eukaryotic initiation factor 2 alpha kinase (eIF2α) pathway protect ovarian cancer cells from metformin-induced apoptosis. Mol. Carcinog..

[81] Dale Rein I., Solberg Landsverk K., Micci F., Patzke S., Stokke T. (2015). Replication-induced DNA damage after PARP inhibition causes G2 delay, and cell line-dependent apoptosis, necrosis and multinucleation. Cell Cycle.

[82] Shi Y., Zhou F., Jiang F., Lu H., Wang J., Cheng C. (2014). PARP inhibitor reduces proliferation and increases apoptosis in breast cancer cells. Chin. J. Cancer Res..

[83] Zheng Y., Zhu J., Zhang H., Liu Y., Sun H. (2018). Metformin inhibits ovarian cancer growth and migration in vitro and in vivo by enhancing cisplatin cytotoxicity. Am. J. Transl. Res..

[84] Sahin I.D., Jönsson J.-M., Hedenfalk I. (2019). Crizotinib and PARP inhibitors act synergistically by triggering apoptosis in high-grade serous ovarian cancer. Oncotarget.

[85] Hijaz M., Chhina J., Dar S., Tebbe C., Al-Wahab Z., Hanna R.K., Rattan R., Munkarah A.R. (2015). Synthetic lethality of PARP inhibitors and metformin in BRCA1 intact ovarian cancer. Gynecol. Oncol..

[86] Musacchio L., Caruso G., Pisano C., Cecere S.C., Di Napoli M., Attademo L., Tambaro R., Russo D., Califano D., Palaia I. (2020). PARP Inhibitors in Endometrial Cancer: Current Status and Perspectives. Cancer Manag. Res..

[87] Han Y., Li C.W., Hsu J.M., Hsu J.L., Chan L.C., Tan X., He G.J. (2019). Metformin reverses PARP inhibitors-induced epithelial-mesenchymal transition and PD-L1 upregulation in triple-negative breast cancer. Am. J. Cancer Res..

[88] Gralewska P., Gajek A., Marczak A., Mikula M., Ostrowski J., Sliwinska A., Rogalska A. (2020). PARP Inhibition Increases the Reliance on ATR/CHK1 Checkpoint Signaling Leading to Synthetic Lethality-An Alternative Treatment Strategy for Epithelial Ovarian Cancer Cells Independent from HR Effectiveness. Int. J. Mol. Sci..

[89] Carmichael J., DeGraff W.G., Gazdar A.F., Minna J.D., Mitchell J.B. (1987). Evaluation of a tetrazolium-based semiautomated colorimetric assay: Assessment of chemosensitivity testing. Cancer Res..

[90] Lopez-Acevedo M., Grace L., Teoh D., Whitaker R., Adams D.J., Jia J., Nixon A.B., Secord A.A. (2014). Dasatinib (BMS-35482) potentiates the activity of gemcitabine and docetaxel in uterine leiomyosarcoma cell lines. Gynecol. Oncol. Res. Pract..

[91] Rogalska A., Gajek A., Lukawska M., Oszczapowicz I., Marczak A. (2018). Novel oxazolinoanthracyclines as tumor cell growth inhibitors-Contribution of autophagy and apoptosis in solid tumor cells death. PLoS ONE.

[92] Rogalska A., Marczak A., Gajek A., Szwed M., Sliwinska A., Drzewoski J., Jozwiak Z. (2013). Induction of apoptosis in human ovarian cancer cells by new anticancer compounds, epothilone A and B. Toxicol. In Vitro.

[93] Rogalska A., Gajek A., Marczak A. (2019). Suppression of autophagy enhances preferential toxicity of epothilone A and epothilone B in ovarian cancer cells. Phytomedicine.

[94] Rogalska A., Gajek A., Marczak A. (2014). Epothilone B induces extrinsic pathway of apoptosis in human SKOV-3 ovarian cancer cells. Toxicol. In Vitro.

[95] Gajek A., Poczta A., Łukawska M., Cecuda- Adamczewska V., Tobiasz J., Marczak A. (2020). Chemical modification of melphalan as a key to improving treatment of haematological malignancies. Sci. Rep..

