# Supplementary material for: Metformin Affects Olaparib Sensitivity through Induction of Apoptosis in Epithelial Ovarian Cancer Cell Lines
Source: Int J Mol Sci. 2021 Sep 29;22(19):10557. doi: 10.3390/ijms221910557 (PMC8508816; doi:10.3390/ijms221910557)
Supplement: Supplementary file 1 [file ijms-22-10557-s001.zip › ijms-1392068-supplementary.pdf]

Table S1. Comparison of the viability and the apoptotic, necrotic and total cells death in SKOV-3 and OV-90 cell lines after 24 h treatment with metformin (20 mM), olaparib (20 μM) and their combination (20 mM + 20 μM).

OV-90								
	**MTT**	**TUNEL**	**Hoechst 33258/Propidium iodide**			**Acridine orange/Ethidium bromide**		
	Viability [% of control]	*TUNEL positive cells [%]*	*Apoptosis [%]*	*Necrosis [%]*	*Total death [%]*	*Apoptosis [%]*	*Necrosis [%]*	*Total death [%]*
C	100	0,9	—	1,8	1,8	—	1,5	1,5
O	94,93	8,2	12,4	4,3	16,7	9	5,3	14,3
M	91,4	7,9	8,5	5,9	14,4	6,7	5,2	11,9
O+M	82,87	17,4	21,7	14,2	35,9	19,1	4,9	24
**SKOV-3**								
C	100	1,2	—	2,3	2,3	0,7	2,8	3,5
O	85,87	22,4	12,7	3,9	16,6	12,7	6,4	19,1
M	85,75	17,9	15,8	9,6	25,4	17	9	26
O+M	55,87	29,7	43,5	19,1	62,6	34,8	17,1	51,9

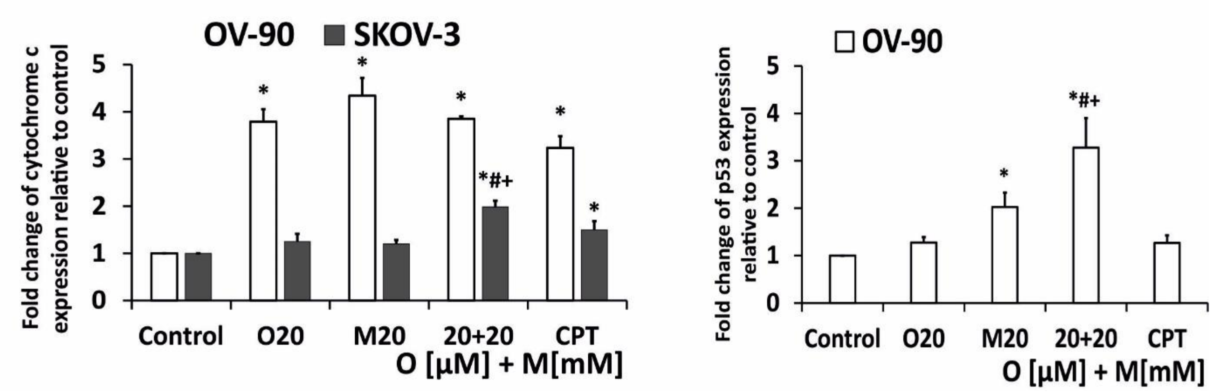

Figure S1. Fold changes of cytochrome c or p53 expression in the OV-90 and SKOV-3 cells treated with O (20 μM), M (20 mM), O (20 μM) + M (20 mM) or CPT (5 μM) for 24 h as measured by western blotting. Data represent the mean ± SD of three independent experiments. * $P < 0.05$ for olaparib, metformin, or a combination of both drugs vs. control cells; # $P < 0.05$ for olaparib vs. combination (O+M); + $P < 0.05$ for metformin vs. combination (O+M).

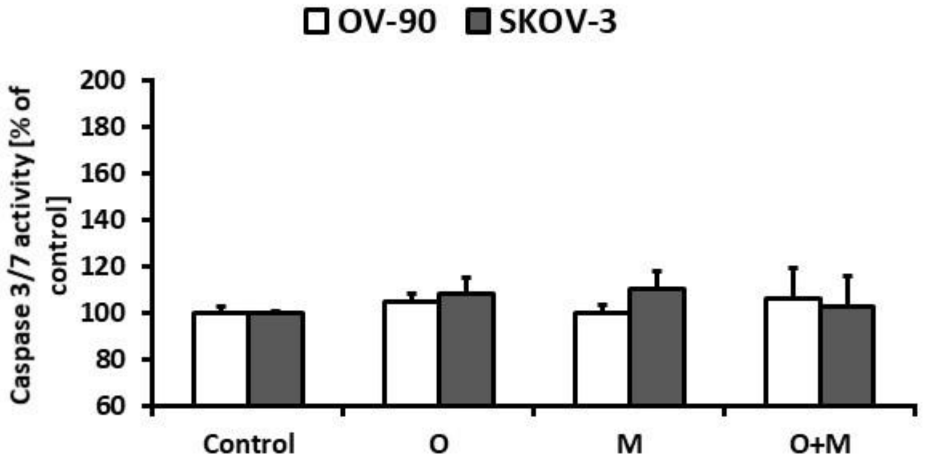

Figure S2. Changes in caspase 3/7 activity in SKOV-3 and OV-90 cells treated with metformin or olaparib and their combination for 24 h in the presence of the caspase inhibitor, Z-FA-FMK. The results are presented as a percentage activity of caspase 3/7, where the fluorescence value of the untreated control was taken as 100%.
